# Burden and determinants of multi-b/tsDMARD failure in psoriatic arthritis

**DOI:** 10.1186/s13075-025-03518-7

**Published:** 2025-03-04

**Authors:** Rebecca H. Haberman, Kyra Chen, Catherine Howe, Seungha Um, Adamary Felipe, Brianna Fu, Stephanie Eichman, Margaret Coyle, Eileen Lydon, Andrea L. Neimann, Soumya M. Reddy, Samrachana Adhikari, Jose U. Scher

**Affiliations:** 1 Department of Medicine, NYU Grossman School of Medicine, New York, NY USA; 2 Department of Population Health, NYU Grossman School of Medicine, New York, NY USA; 3 Ronald O. Perelman Department of Dermatology, NYU Grossman School of Medicine, New York, NY USA; 4 Colton Center for Autoimmunity, NYU Grossman School of Medicine, New York, NY USA

**Keywords:** Psoriatic arthritis, Biologics, Outcomes, Therapeutics, Difficult to treat

## Abstract

**Objectives:**

Despite significant therapeutic advances in psoriatic arthritis (PsA), many patients do not achieve remission and cycle through multiple biologic (b)- or targeted synthetic (ts)- DMARDs. Identifying the underlying reasons for repetitive therapeutic failure remains a knowledge gap. Here we describe prescribing patterns and characteristics of PsA patients with multi-b/tsDMARD failure at the NYU Psoriatic Arthritis Center.

**Methods:**

Nine hundred sixty PsA patients were enrolled in an observational, longitudinal registry. Demographics, medical history, medication use, and psoriatic disease phenotype were collected. Multi-b/tsDMARD failure was defined as requiring ≥ 4 b/tsDMARDs.

**Results:**

Seven hundred twenty-five patients (75%) used ≥ 1 b/tsDMARD during their disease course. The initial b/tsDMARDs prescribed were predominately anti-TNF agents. 166 (17%) patients had multi-b/tsDMARD failure. Compared to those requiring 1 b/tsDMARD, female sex (OR 2.3; 95%CI 1.4–3.8), axial disease (OR 2.1; 95% CI 1.2–3.6), depression (OR 2.0; 95%CI 1.1–3.7), and obesity (OR 1.7; 95%CI 1.0–2.8) were risk factors for multi-b/tsDMARD failure disease after adjustment for age, disease duration, sex, depression, smoking, obesity, and skin severity. Patients with multi-b/tsDMARD failure PsA also had increased disease activity at their clinical visit (i.e., swollen joint count, p = 0.005).

**Conclusion:**

In this cohort, 17% patients with PsA experienced multi-b/tsDMARD failure. These patients were more likely to be female, obese, and have higher rates of axial involvement and depression, along with higher active disease activity. This highlights the inflammatory and non-inflammatory drivers of multiple therapeutic failures, underscoring the need for precision medicine strategies and potential non-pharmacologic adjuvant therapies for patients with PsA to improve outcomes and quality of life.

**Supplementary Information:**

The online version contains supplementary material available at 10.1186/s13075-025-03518-7.

## Introduction

Psoriatic arthritis (PsA) is a chronic, immune-mediated inflammatory arthritis associated with cutaneous psoriasis. It is a heterogenous disease involving multiple domains including peripheral joints, entheses, skin, nails, and axial spine that can lead to erosive and deforming disease if left untreated [1]. There has been significant progress in psoriatic therapeutics over the last two decades with the advent of biologic (b)- and targeted synthetic (ts)- disease modifying agents (b/tsDMARDs), first with tumor necrosis factor inhibitors (TNFis) and most recently with the introduction of interleukin (IL) −17 and −23 and Janus kinase (JAK) inhibitors. While many patients achieve skin clearance or almost clearance with these strategies, up to 50% of patients with PsA do not experience significant synovio-enthesial improvement by blocking the same pathways [2–4]. This can lead to patients with PsA cycling quickly and frequently through multiple b/tsDMARDs. In fact, studies across the US and Europe have shown relatively low persistence rates of psoriatic therapeutics [5–9]. This is particularly problematic since it has been shown that with each successive therapy, the rates of remission decline, with some studies showing remission rates as low as 20% in just the second and third medication courses [5].

Given this, the concept of difficulty-to-treat (D2T) PsA has become an important topic in the field [10–12]. While organizations such as GRAPPA and EULAR are working to delineate this term, there are no current consensus definitions. In fact, these patients may reflect two distinctive, but not mutually exclusive, phenotypes: (1) those with active inflammation and immune system dysfunction that is refractory to the currently available treatments and (2) those with comorbidities (i.e., depression) and non-inflammatory symptoms (i.e., chronic pain syndromes) that make remission difficult to achieve. However, little is known about these patients and what deem them susceptible to failing multiple b/tsDMARD.

Here we examined the prescribing practices in a large, urban combined psoriasis-psoriatic arthritis center and describe the characteristics of patients with multi-b/tsDMARD failure PsA (i.e., those requiring 4 or more b/tsDMARD therapeutics) compared to those on their first b/tsDMARD.

## Methods

### Study design and population

960 consecutive adult patients meeting CASPAR criteria [13] for PsA were enrolled in a longitudinal, prospective, cohort registry from the NYU Psoriatic Arthritis Center (NYU PAC) and associated outpatient clinics and practices, including Bellevue Hospital. Patients were enrolled from January 2014 to September 2024. The study was approved by the NYU Institutional Review Board (s20-00084) with waived written consent from individual participants as all data was collected in a deidentified manner.

### Data and outcomes

Demographics, comorbidities, psoriatic disease phenotype and activity, current and past medication use, and patient reported outcomes were extracted from the electronic medical record (EMR) and entered into a REDCap [14] database. EMR data included clinical notes from a PsA-specific template designed to capture discrete disease characteristics and phenotypes. All patients were evaluated by dedicated rheumatologists with expertise in psoriatic disease.

Patients were defined as having multi-b/tsDMARD failure disease if they had used 4 or more b/tsDMARDs over their disease course, regardless of current use or mechanism of action (MOA). A secondary definition of multi-b/tsDMARD failure disease (requiring 3 or more b/tsDMARDs) was also used. b/tsDMARDs included agents approved for psoriasis and/or PsA targeting TNF (adalimumab, etanercept, golimumab, certolizumab, infliximab), IL-12/23 (ustekinumab), IL-17 (secukinumab, ixekizumab, brodalumab), IL-23 (guselkumab, risankizumab, tildrakizumab), JAK (tofacitinib, upadacitinib), IL-17AF (bimekizumab), and tyrosine kinase 2 (Tyk2, deucravacitinib). Conventional synthetic (cs) DMARDs and phosphodiesterase 4 (PDE4) inhibitors included: methotrexate, sulfasalazine, leflunomide, and apremilast. Of note, while apremilast is considered a tsDMARD, for purposes of this analysis, it was excluded as the overall efficacy on joint outcomes is significantly lower than the above b/tsDMARDs [15, 16]. Medication discontinuation was considered a “failure” regardless of the reason, including patient’s voluntary withdrawal (e.g., stopping because they felt well, concern for possible future side effects). Medication use and information regarding reasons for discontinuation were by patient report and EMR; patients were able to report “unknown” for any variable. Primary medication failure was defined as failure due to skin-, joint-domain/s, or both.

All patient characteristics and physical exams analyzed were as of most recent in-person clinical encounter at the NYU Langone Health system, regardless of timepoint in disease duration or treatment. Axial disease was defined as having current or prior inflammatory back pain with either X-ray and/or MRI findings consistent with sacroiliitis and/or spine involvement. Other psoriatic disease domain and characteristics (i.e., enthesitis, dactylitis, nail disease, area of psoriasis involvement) were considered positive if there was any history of involvement over the disease course. Comorbidities (with the exception of obesity), were by patient report and medical history, regardless of current activity. Obesity was defined as a body mass index (BMI) of ≥ 30 at the time of the assessed visit.

### Statistical analysis

Baseline characteristics of study participants were summarized using frequency and proportion for categorical variables and mean and standard deviations (SD) and/or median and interquartile ranges (IQRs) for continuous variables. Statistical comparisons between groups were performed using independent two-sample t tests, ANVOA, Fischer’s exact test, and Mann–Whitney U tests to test for group differences as appropriate. For disease activity measures, both parametric and non-parametric tests were performed for sensitivity analyses. Logistic regression was used to estimate associations (odds ratios, OR and corresponding 95% confidence intervals (CI)) with adjustment for age, sex, disease duration, smoking status, obesity, depression, and skin severity. Changes in treatment persistence over time were estimated using the Kaplan–Meier Method. Cox proportional hazards regression models were used to estimate the hazard ratios (HRs) and 95% CIs. The proportional hazards assumption was tested visually and by testing for any significant interaction with time. Data were analyzed using statistical software SPSS [17].

## Results

### Cohort description and characteristics

**Fig. 1:**
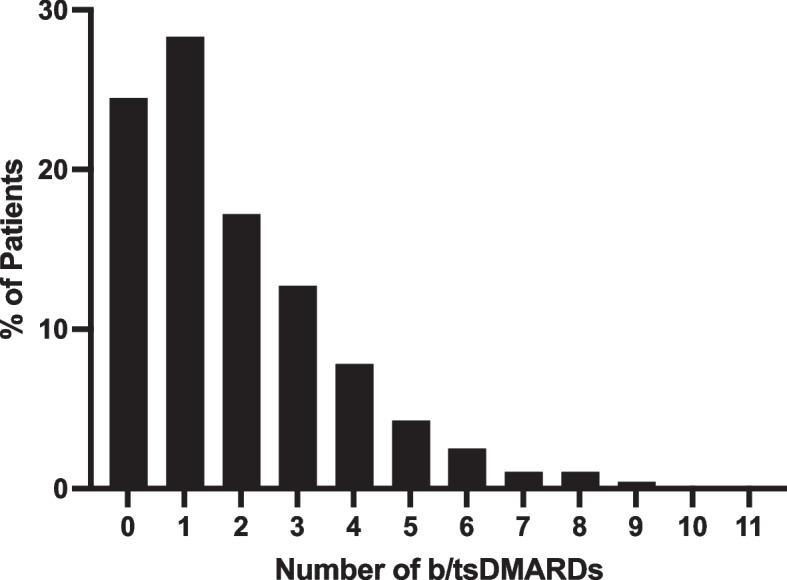
Number of biologic and targeted synthetic disease modifying anti-rheumatic drugs (b/tsDMARDs) exposures

The NYU PAC cohort consisted of 960 unique individuals who were followed for an average of 30.03 months (SD 31.84) (Supplementary Table S1). They had a median age of 49 years (IQR 37–61), were 51.46% male, and 82.8% White with a median disease duration of 7 years (IQR 3–13). 725 of these patients (75.5%) were exposed to at least 1 b/tsDMARD throughout the course of their disease (Fig. 1).

### b/tsDMARD prescribing patterns and retention

**Fig. 2:**
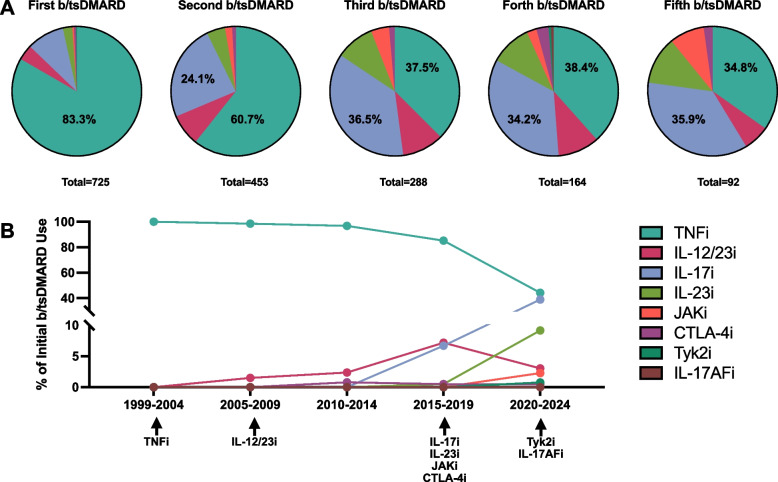
b/tsDMARD prescription patterns. **A** Mechanism of action of by b/tsDMARD exposure. **B** Mechanism of action of the first b/tsDMARD exposure by time period. Arrows and medications denote the time period where the first medication of that MOA was FDA approved for the indication of either psoriasis or psoriatic arthritis. TNFi denotes tumor necrosis factor inhibitor, IL- 12/23i interleukin 12/23 inhibitor, IL-17i interleukin 17 inhibitor, IL-23i interleukin 23 inhibitor, JAKi janus kinase inhibitor, CTLA-4i cytotoxic T-lymphocyte associated protein 4 inhibitor, Tyk2i tyrosine kinase inhibitor, IL-17AFi interleukin 17AF inhibitor

**Fig. 3:**
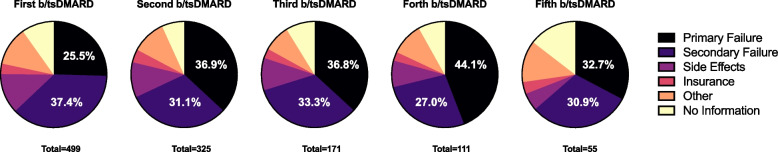
Reason for discontinuing b/tsDMARD by exposure

TNFis were the first- and second-line b/tsDMARD agents used in 83.3% (604/725) and 60.7% (275/453) of patients respectively (Fig. 2A). TNFis and IL-17is were used equally as third, fourth-, and fifth-line agents. From 1999 to 2019, TNFis were the predominant first-line agents, with IL-17is reaching similar levels in the time period between 2020 and 2024 (Fig. 2B). The most common reason for discontinuation of the first b/tsDMARD was secondary failure (187/499, 37.5%), followed by primary failure (127/499, 25.5%) (Fig. 3). Primary failure was inclusive of primary skin failure (36/499, 7.2%), primary joint failure (31/499, 6.2%), and failure of both skin and joints (60/499, 12.0%) (Supplementary Figure S1). For later exposures, the most common reason was primary failure (up to 44.1% in the fourth therapeutic course).

**Fig. 4:**
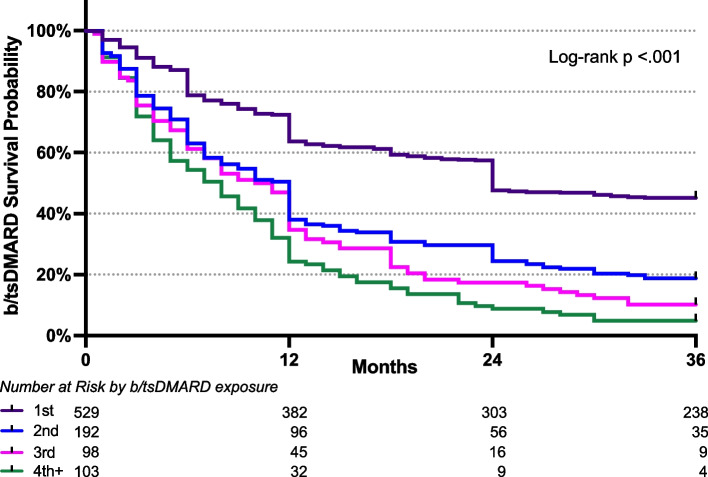
Kaplan–Meier Estimates of b/tsDMARD Persistence by Exposure. Persistence was defined as the time from therapy initiation to discontinuation. Patients exposed to 4 or more treatments were grouped into the 4^th^+ cohort

Of the b/tsDMARD-exposed population (*n* = 725), 288 (39.7%) had been prescribed with 3 or more therapies, 166 (22.9%) patients were prescribed 4 or more, and 91 (12.6%) had seen 5 or more different b/tsDMARDs (Fig. 1). On average, patients were exposed to a median of 1 (IQR 1–2) MOA with 125 individuals (18.6%) exposed to 3 or more MOAs. Using Kaplan–Meier survival analysis, the persistence rate for the first b/tsDMARD used was 72.2% at year 1, 57.5% at year 2, and 45.2% at year 3 with an average survival estimate of 22.9 months (95%CI 21.7–24.0) (Fig. 4**, **Supplementary Table 2). Women were more likely to discontinue their first b/tsDMARD at year 1 (HR 1.74, 95%CI 1.25–2.42, *p* = 0.001) and year 3 (HR 1.64, 95%CI 1.30–2.07,* p* < 0.001) compared to men (Supplementary Figure S2, Supplementary Table 3). Although women in this cohort were more likely to be obese (37.4% vs 29.1%, *p* = 0.025) and have current or past depression (21.9% vs 14.2%, *p* = 0.009), this remained true when adjusting for depression, obesity, and smoking status (HR 1.49, 95%CI 1.05–2.11, *p* = 0.025 at 1 year and HR 1.48, 95%CI 1.16–1.89,* p *= 0.002 at 3 years). Medication persistence was not affected by age, obesity, depression, smoking status, axial domain involvement, dactylitis, or peripheral erosions (Supplementary Table 3).

Medication survival decreased with each subsequent exposure with average survival estimates of 14.9 months (95%CI 13.1–16.6), 12.6 months (95% CI 10.5–14.8), and 10.1 months (95%CI 8.3–11.8) for second, third, and fourth or more exposure respectively. Additionally, subsequent exposures were more likely to discontinue the drug by year 3 compared to the first exposure (HR 2.08, 95%CI 1.71–2.53, *p *< 0.001 for second; HR 2.96, 95%CI 2.04–3.30, *p *< 0.001 for third; HR 3.34, 95%CI 2.64–4.21, *p* < 0.001 for forth or more).

### Characteristics and risk factors of multi-b/tsDMARD disease

**Fig. 5:**
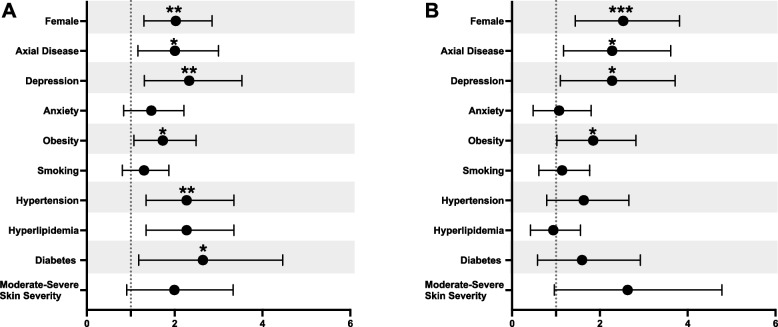
Risk estimates of multi-b/tsDMARD failure psoriatic arthritis. Odds ratios are unadjusted (**A**) and adjusted (**B**) for disease duration, age, sex, depression, smoking status, obesity, and skin severity

Individuals with multi-b/tsDMARD failure disease (exposed to 4 or more b/tsDMARDs) were compared to those who had only been exposed to 1 b/tsDMARD (Table 1). Mulit-failure patients were older (median 55 years vs 45 years, p < 0.001) and had a longer disease duration (median 11 years vs 6 years, *p* < 0.001). They were more likely to be female (60% vs 43%, *p* = 0.001), have axial involvement (26% vs 16%, *p *= 0.013), and have a history of depression (25% vs 13%, p = 0.003), hypertension (31 vs 17%, *p* = 0.001) and type 2 diabetes mellitus (13% vs 6%, p = 0.015). While BMI was similar between groups, the proportion of patients classified as obese was higher in the multi-b/tsDMARD failure patients (37% vs 26%, p = 0.022). Depression conferred the highest risk of multi-b/tsDMARD failure disease (OR 2.15, 95%CI 1.31–3.53), followed by female sex (OR 1.93, 95%CI 1.30–2.85), presence of axial disease (OR 1.86, 95%CI 1.16–3.00), and obesity (OR 1.63, 95%CI 1.07–2.49) (Fig. 5A, Supplementary Table 4). These risks remained significant even after adjusting for age, disease duration, sex, depression, smoking status, obesity, and skin severity (Fig. 5B, Supplementary Table 4).

**Table 1:** Characteristics of patients with multi-b/tsDMARD failure disease compared to those who have been exposed to 1 b/tsDMARD

Characteristics	All (*n* = 438)	1 b/tsDMARD (*n* = 272)	Multi-b/tsDMARD Failure PsA (*n* = 166)	*p*-value
***Demographics***				
Age- median (IQR)	49 (37–60.5)	45 (35–59)	55 (40–63.25)	< 0.001
Female- n (%)	217 (49.54)	118 (43.38)	99 (59.64)	0.001
Race/Ethnicity- n (%)^a^				
Asian	0 (0.00)	0 (0.00)	0 (0.00)	0.339
Black	41 (9.36)	30 (11.03)	11 (6.63)	
White	0 (0.00)	0 (0.00)	0 (0.00)	
Other	8 (1.83)	5 (1.84)	3 (1.81)	
Hispanic	363 (82.88)	221 (81.25)	142 (85.54)	
***Psoriatic Disease and Treatment Timeline – median (IQR)***				
Age PsO Onset^b^	25 (17–36)	25.5 (18–35.75)	23 (14–37)	0.145
Age PsA Onset	37 (28–48)	36 (27–47)	39 (28.5–50)	0.488
PsO to PsA Transition (years)	8 (2–17)	8 (2–15)	9 (2–21)	0.171
PsA Diagnosis Delay (years)	1 (0–2)	1 (0–2)	0 (0–2)	0.079
Disease Duration (years)	7 (4–14)	6 (3–12)	11 (5.25–18.75)	< 0.001
Time to First Biologic (years)	1 (0–2.75)	1 (0–3)	0.5 (0–2)	0.304
Year of First Biologic	2016 (2011–2019)	2016 (2013.75–2020)	2013 (2006–2017)	< 0.001
Number of b/tsDMARDs	1 (1–4)	1 (1–1)	5 (4–6)	–
Number of MOAs	1 (1–2)	1 (1–1)	3 (2–3)	–
csDMARD^c^ use ever—n(%)	273 (58.33)	141 (51.84)	132 (79.52)	< 0.001
***Psoriatic Disease Phenotype- n (%)***				
Imaging Erosions	110 (25.11)	61 (22.43)	49 (29.52)	0.112
Peripheral Deformities	42 (9.59)	29 (10.66)	13 (7.83)	0.404
Enthesitis	151 (34.47)	85 (31.25)	66 (39.76)	0.072
Dactylitis	125 (28.54)	73 (26.84)	52 (31.32)	0.328
Axial Disease	86 (19.63)	43 (15.81)	43 (25.90)	0.013
Scalp Psoriasis	256 (58.45)	161 (59.19)	85 (51.20)	0.691
Inverse Psoriasis	69 (15.75)	42 (15.44)	27 (16.27)	0.893
Nail Involvement	208 (47.49)	124 (45.59)	84 (50.60)	0.325
***Comorbidities- n(%)***				
Uveitis	14 (3.20)	7 (2.57)	7 (4.22)	0.405
Inflammatory Bowel Disease	11 (2.51)	4 (1.47)	7 (4.22)	0.112
Depression	77 (17.58)	36 (13.24)	41 (24.70)	0.003
Anxiety	82 (18.72)	46 (16.91)	36 (21.69)	0.256
ADHD	14 (3.20)	7 (2.57)	7 (4.22)	0.405
Obesity	133 (30.40)	71 (26.10)	62 (37.35)	0.022
Hypertension	98 (22.37)	47 (17.28)	51 (30.72)	0.001
Hyperlipidemia	87 (19.86)	49 (18.01)	38 (22.89)	0.220
Diabetes Mellitus	39 (8.90)	17 (6.25)	22 (13.25)	0.015
Fibromyalgia	8 (1.83)	5 (1.84)	3 (1.81)	> 0.999
Current/Former Smoker	133 (30.40)	78 (28.68)	55 (33.13)	0.337
***Disease Activity – mean (SD)/ median (IQR)***				
Tender Joint Count	1.95 (3.83) / 0 (0–2)	1.52 (3.44)/ 0 (0–1)	2.67 (4.31)/ 1(0–3)	0.004/ < 0.001
Swollen Joint Count	1.04 (2.46) / 0 (0–1)	0.78 (1.95)/ 0 (0–0)	1.48 (3.09) / 0 (0–1.5)	0.007/0.005
% Psoriasis BSA	1.37 (4.59) / 0.5 (0–1)	1.02 (2.02)/ 0.5 (0–1)	1.91 (6.87) / 0.5 (0–1)	0.060/0.694
Moderate to Severe PsO^d^	41 (9.36)	20 (7.35)	21 (12.65)	0.127
Body Mass Index—mean (SD)	29.31 (17.22)	29.16 (21.24)	29.54 (7.07)	0.828
Physician Global	2.19 (1.63) / 2 (1–3)	1.81 (1.46) / 1.5 (1–2.38)	2.76 (1.69) / 2.5 (1.5–2.5)	< 0.001/ < 0.001
RAPID 3—mean (SD)	10.32 (6.31)	8.36 (5.74)	12.91 (6.14)	< 0.001
Erythrocyte Sedimentation Rate	22.05 (21.00) / 16 (7–32)	17.46 (17.87) / 10 (5–24)	29.20 (23.49) / 22.5 (14–36)	< 0.001/ < 0.001
C-Reactive Protein	4.77 (8.37) / 2 (0.7–5)	4.05 (8.51) / 1.5 (0.6–4.55)	5.95 (8.05) / 2.45 (1–6.25)	0.102/0.016
Current Use of csDMARD- n(%)	127 (29.00)	63 (23.16)	64 (38.55)	< 0.001
Current Use of b/tsDMARD- n(%)	351 (80.14)	223 (81.99)	128 (77.11)	0.173

^a^Patients may identify as more than one race/ethnicity

^b^*PsO* denotes skin psoriasis, *PsA* psoriatic arthritis, *MOA* mechanism of action, *ADHD* attention deficit hyperactivity disorder, *BSA *body surface area, *b/tsDMARD* biologic and targeted synthetic disease modifying antirheumatic agents, *csDMARD* conventional synthetic DMARD

^c^csDMARD also includes PDE4 inhibitors

^d^Moderate to severe PsO is BSA ≥ 3%

To account for patients only exposed to 1 b/tsDMARD due to recent diagnoses, we performed a sensitivity analysis comparing those with multi-b/tsDMARD failure disease to those with 1 b/tsDMARD exposure, restricted to patients with at least 5 years of disease duration. Depression (OR 2.01, 95%CI 1.10–3.66), female sex (OR 1.99, 95%CI1.26–3.15), obesity (OR 2.83, 95%CI 1.63–4.93), and axial disease (OR 1.89, 95%CI 1.07–3.36) remained significant (Table S5-6, Supplementary Figure S3). Additionally, having moderate to severe skin disease (greater than 3% BSA) was associated with higher risk of multi-b/tsDMARD failure (OR 2.78, 95%CI 1.13–6.85). When using 3 or more b/tsDMARDs as a secondary definition of multi-b/tsDMARD failure disease, these patients were still more likely to be older (median 51 vs 45, *p* = 0.005) with a longer disease duration (median 9 years vs 6 years, *p* < 0.001), be female (53% vs 43%, *p* = 0.022), and have axial involvement (23% vs 15%, *p* = 0.041), depression (24% vs 13%, *p *< 0.001), obesity (35% vs 26%, *p *= 0.024), and hypertension (27% vs 17%, *p *= 0.008). They were also more likely to have entheseal involvement (40% vs 31%, *p *= 0.048) but less likely to have peripheral deformities (5% vs 11%,* p* = 0.018) (Supplementary Table S7-8, Supplementary Figure S4). In comparing use of different MOAs, patients requiring 3 or more MOAs compared to 1 MOA had a longer disease duration (median 9 years vs 7 years, *p* = 0.006) and a greater proportion had moderate to severe psoriasis (15% vs 9%, *p* = 0.036) (Supplementary Table S9).

At the most recent clinical encounter at the NYU PAC, multi-b/tsDMARD failure patients had more active joint disease as evidenced by higher mean tender and swollen joints counts, physician global scores, RAPID3 scores, and erythrocyte sedimentation rates (although C-reactive protein levels were similar). The same was seen when adjusting for age and sex (*p *= 0.015,* p* = 0.010, *p* < 0.001,* p* < 0.001, *p* < 0.001, p = 0.105 respectively). We also performed a sensitivity analysis using non-parametric testing. All results remained, with the exception of CRP, which was now statistically higher in the multi-b/tsDMARD failure patients (*p *= 0.016). Additionally, when limiting the analysis to only patients currently on a b/tsDMARD at the most recent visit (n = 350), the clinical exam differences remained the same (Supplementary Table S10).

## Dissussion

The heterogeneity of PsA can make it intrinsically difficult to select therapeutic agents that lead to an adequate clinical response. Additionally, PsA is associated with multiple comorbidities (including cardiovascular, gastrointestinal, and mental health disorders) that can interfere with (and/or prevent) resolution of symptoms. Despite the dramatic increase in the number of pathway-targeted therapies in psoriatic disease, patients with PsA often have low medication persistence, cycle quickly through multiple medications, and can ultimately be left with few treatment options. Therefore, it is imperative to better understand prescribing patterns, how and why patients respond to currently available strategies, and identify those who do not respond, so that appropriate interventions can be timely deployed.

In a cross-sectional analysis of a large combined, psoriasis-PsA setting, we found that over 75% of patients were exposed to at least one b/tsDMARD throughout their disease course. By far the most commonly prescribed first-line b/tsDMARDs were TNFis. However, this is likely reflective of the availability of TNFis, which had an earlier approval date, and for a long time had better insurance coverage, compared to other MOAs. Underlining this point, is the rise of IL-17is as first line agents since their approval in 2015, almost reaching the level of TNFis in our cohort between 2020–2024.

In line with previous studies [5–9], we found each successive medication exposure was associated with decreasing medication persistence. The estimated drug survival dropped from 23 to 15 months when moving from the first to second exposure. A significant proportion of the NYU PAC patient population (17%) eventually required the use of 4 or more courses of different medications (i.e., multi-b/tsDMARD disease). By this fourth exposure, estimated mean medication survival was 10.1 months with only 32% of patients remaining of the medication at 1 year. This recapitulates the known fact that as patients cycle away from their first b/tsDMARD, the chances of finding an effective therapy further declines, emphasizing the importance of better understanding the phenotype and immunoendotypes of these multi-b/tsDMARD failure patients.

We applied the term *multi-b/tsDMARD PsA *to describe our studied population as we recognize that formal consensus definitions are currently under development for the term “D2T” by both GRAPPA and EULAR [11, 18]. Past cohort studies have generally used failure of at least 2 b/tsDMARDs to classify patients as D2T based on an extrapolation of the EULAR rheumatoid arthritis definition [19]. In this study, we focused on a population of patients who had failed 4 or more b/tsDMARDs for a stricter and more conservative analysis. This is also an important clinical timepoint, where further therapeutic options decrease significantly, patients and health care providers often feel frustrated, and persistence on subsequent medications (as well as clinical response) is exceptionally low.

Here we present the first US-based cohort (and largest to our knowledge) of patients with multi-b/tsDMARD failure in PsA. Importantly, and despite using different definitions, European/Asian cohorts have found similar results in terms of the proportion of “D2T” patients as well as the risk factors of female sex, obesity, and the presence of axial disease. A Greek cohort of 467 patients, found that 16.5% were considered D2T (defined as having failed at least 1 csDMARD and at least 2 b/tsDMARDs of different MOAs and with current moderate disease activity), which was very similar to our 17.3% prevalence of multi-b/tsDMARD failure patients. They found that patients considered to be D2T were more likely to have extensive skin psoriasis, a higher BMI, history of inflammatory bowel disease, and, akin to our findings, were more likely to be female and have axial disease [20]. Additionally, in terms of skin involvement, a cohort from University of San Francisco looked at 222 patients with skin psoriasis only and found that duration of psoriasis and erythrodermic psoriasis were risk factors for the use of 3 or more b/tsDMARDs compared to 1 [21]. While we did not find any relationship with skin severity and involvement in our main analysis, over 90% of the patients in our cohort have mild cutaneous involvement which may limit our ability to identify any differences in this variable.

Additionally, in a French cohort of 150 individuals with PsA the investigators found that D2T patients (defined as failing at least 2 b/tsDMARDs with different MOAs) were also more likely to have higher rates of axial disease [22]. A Turkish Cohort of 171 patients, defined similarly to the French cohort, found that 19.3% of patients were D2T and had higher rates of fibromyalgia and diabetes, with a higher number of overall comoribidties [23]. Lastly, an Italian cohort of 106 patients found that those who were D2T had a higher BMI, higher rates of fibromyalgia, and an increased delay in time to first b/tsDMARD (when defined as failing least 1 csDMARD and 2 b/tsDMARDs, having evidence of disease activity, and being perceived as having problematic to treat disease by the rheumatologist and/or patient) [24].

Female sex, depression, and obesity are likely interconnected and independent risk factors for multi-b/tsDMARD failure. Female patients have been shown to have decreased rates of response to therapy and reduced medication persistence [25–27]. In our study, we again demonstrated decreased drug persistence, but also an increased risk of multi-b/tsDMARD failure, even when controlling for possible confounding factors like depression and obesity. The reasons behind these differences are unclear but may include hormonal and biologic sex-based differences in addition to higher rates of depression, obesity, and fibromyalgia. Depression itself increased the risk of multi-b/tsDMARD failure disease by over 2 times in our cohort, whether or not depression was currently active. Previous studies have shown that patients with concomitant depression (regardless of sex) have decreased remission rates, driven by patient reported pain and tender joint counts rather than physician or laboratory confirmed inflammation [28–30]. Therefore, “failure” of patients with depression may be due to differences in pain perception rather than continued active inflammation from PsA.

Patients with multi-b/tsDMARD failure disease were also more likely to be obese and have axial domain involvement. Obesity has been linked to decreased remission rates and medication response [31, 32], possibly due to its impact on the therapeutic pharmacokinetics as well as the fact that obesity and adipose tissue are associated with a chronic state of low grade inflammation [33, 34]. Additionally, previous studies have found that patients with PsA and axial domain involvement (whether currently active or inactive) are more likely to have a history of depression, decreased rates of minimal disease activity, and worse patient reported outcomes compared to those without axial involvement, which may account for the higher risk of multi-b/tsDMARD failure disease [35].

In our study, multi-b/tsDMARD failure patients also demonstrated higher disease activity, even in physician-assessed outcomes (swollen joint count) and acute phase reactants at the time of evaluation. Of note, however, swollen joint count was low overall in both groups (0.8 and 1.5 joints), consistent with the oligoarticular nature of PsA patients in real-world practices. This indicates that multi-b/tsDMARD failure patients likely have a degree of remaining inflammatory disease activity.

We acknowledge several limitations in our study. First, much of the data relies on patient report and therefore is subject to recall bias. In particular, the order of medication use, duration on each medication, and reason for failure may have been reported years after medication discontinuation. Up to 10% of our patients reported reason for failure as unknown/do not remember. For those seen within the Epic EMR, these variables were checked against physician notes and medication orders, but this was not available in all cases. Second, we recognize that our multi-b/tDMARD failure patients are heterogenous in the reasons for “failure” and yet we treat all reasons for discontinuation as equal. For example, a primary failure is considered the same as a switch for insurance reasons or voluntary withdrawal. However, while this presents a limitation, it is also reflective of real-world situations and patient attitudes. Third, patients are evaluated at different points in their disease state and treatment course.

Given these findings, the use of multiple consecutive b/tsDMARDs is likely driven by both inflammatory and non-inflammatory factors. Future research is needed to parse out these populations as different treatment strategies are still needed. For those with inflammatory states resistant to currently available treatments, strategies such as new or combined molecular targets and even prevention of disease are needed [36, 37]. However, sex, depression, obesity, and even some aspects of axial domain involvement are factors that cannot be directly addressed with the use of immunomodulators. Therefore, continually switching b/tsDMARDs will most likely remain an ineffective strategy. Early adoption of adjuvant therapies, such as weight loss interventions, anti-depressants, cognitive behavior therapy, and/or physical therapy may be the key to overall symptomatic improvement and achievement of better outcomes in PsA.

## Supplementary Information

Supplementary Material 1

## Data Availability

Data will be made available upon request to the authors.
