# Supplementary material for: Burden and determinants of multi-b/tsDMARD failure in psoriatic arthritis
Source: Arthritis Res Ther. 2025 Mar 4;27:46. doi: 10.1186/s13075-025-03518-7 (PMC11877731; doi:10.1186/s13075-025-03518-7)
Supplement: Supplementary file 1 — Supplementary Material 1 [file 13075_2025_3518_MOESM1_ESM.docx]

**Supplement to:**

Burden and Determinants of Muti-b/tsDMARD Failure in Psoriatic Arthritis

Rebecca H. Haberman, Kyra Chen, Catherine Howe, Seungha Um, Adamary Felipe, Brianna Fu, Stephanie Eichman, Margaret Coyle, Eileen Lydon, Andrea L. Neimann, Soumya M. Reddy, Samrachana Adhikari, Jose U. Scher

**Table of Contents:**

Tables

| Table S1. Baseline characteristics of total patient population | 2 |
| --- | --- |
| Table S2. Survival estimates by b/tsDMARD exposure | 4 |
| Table S3. Hazard ratio for first b/tsDMARD persistence | 4 |
| Table S4. Regression models for multi-failure vs 1 b/tsDMARD exposure | 5 |
| Table S5. Baseline characteristics of patients requiring 1 b/tsDMARD (with at least 5 years of disease) compared to multi-failure patients | 6 |
| Table S6. Regression models for multi-b/tsDMARD failure vs 1 b/tsDMARD exposure with ≥5 years of disease duration | 8 |
| Table S7. Baseline characteristics of patients requiring 1 b/tsDMARD compared to those requiring 3+ b/tsDMARDS | 9 |
| Table S8. Regression models for patients requiring 3+ b/tsDMARDs compared to those requiring 1 b/tsDMARD | 11 |
| Table S9. Baseline characteristics of patients by mechanism of action | 12 |
| Table S10. Disease activity of patients requiring 1 b/ts DMARD compared to multi-b/tsDMARD failure patients (in those currently on b/tsDMARD at time of visit) | 14 |

Figures

| Figure S1. Figure S1. Reason for discontinuing b/tsDMARD by exposure (including type of primary failure) | 15 |
| --- | --- |
| Figure S2. Kaplan-Meier estimates of first b/tsDMARD persistence by sex | 16 |
| Figure S3. Risk estimates of multi-b/tsDMARD failure psoriatic arthritis compared to those with 1 b/tsDMARD exposure and at least 5 years of disease | 17 |
| Figure S4. Risk estimates of requiring 3+ b/tsDMARDs compared to 1 b/tsDMARD. | 17 |

**Table S1. Baseline characteristics of total patient population**

| Characteristics | All  (n=960) | No b/tsDMARD exposure (n=235) | + b/tsDMARD exposure (n=725) | p-value |
| --- | --- | --- | --- | --- |
| *Demographics* |  |  |  |  |
| Age- median (IQR) | 49 (37-61) | 49 (37-60) | 48.5 (37-61) | 0.701 |
| Female- n (%) | 466 (48.54) | 114 (48.51) | 352 (48.55) | 0.213 |
| Race/Ethnicity- n (%)* |  |  |  | 0.927 |
| Asian | 80 (8.33) | 20 (8.51) | 60 (8.28) |  |
| Black | 17 (1.77) | 5 (2.13) | 12 (1.66) |  |
| White | 795 (82.81) | 193 (82.13) | 602 (83.03) |  |
| Other | 68 (7.08) | 17 (7.23) | 51 (7.03) |  |
| Hispanic | 74 (7.71) | 14 (5.96) | 60 (8.28) | 0.456 |
| *Psoriatic Disease and Treatment Timeline – median (IQR)* | | | |  |
| Age PsO Onset^ | 25 (17-38) | 29 (19-41.75) | 25 (16-35) | 0.001 |
| Age PsA Onset | 38 (29-49) | 42 (32-52) | 37 (28-48) | <0.001 |
| PsO to PsA Transition (years) | 8 (2-17) | 8 (2-14.75) | 8 (2-17) | 0.816 |
| PsA Diagnosis Delay (years) | 1 (0-2) | 1 (0-3) | 1 (0-2) | 0139 |
| Disease Duration (years) | 7 (3-13) | 3 (0-8) | 8 (4-14) | <0.001 |
| Time to First Biologic (years) | --- | --- | 1 (0-3) | --- |
| Number of b/tsDMARDs | --- | --- | 2 (1-3) | --- |
| Number of MOAs | --- | --- | 1 (1-2) | --- |
| *Psoriatic Disease Phenotype- n (%)* | |  |  |  |
| Imaging Erosions | 222 (23.13) | 42 (17.87) | 180 (24.83) | 0.028 |
| Peripheral Deformities | 76 (7.92) | 16 (6.81) | 60 (8.28) | 0.578 |
| Enthesitis | 345 (35.94) | 90 (38.30) | 255 (35.17) | 0.296 |
| Dactylitis | 273 (28.44) | 73 (31.06) | 200 (27.59) | 0.318 |
| Axial Disease | 172 (17.92) | 30 (12.77) | 142 (19.59) | 0.019 |
| Scalp Psoriasis | 592 (61.67) | 141 (60.00) | 426 (58.76) | 0.939 |
| Inverse Psoriasis | 162 (16.88) | 48 (20.43) | 114 (15.72) | 0.109 |
| Nail Involvement | 482 (50.21) | 113 (48.09) | 369 (50.90) | 0.499 |
| *Comorbidities- n(%)* |  |  |  |  |
| Uveitis | 29 (3.02) | 5 (2.13) | 24 (3.31) | 0.510 |
| Inflammatory Bowel Disease | 21 (2.19) | 3 (1.28) | 18 (2.48) | 0.440 |
| Depression | 162 (16.88) | 32 (13.62) | 130 (17.93) | 0.134 |
| Anxiety | 180 (18.75) | 37 (15.74) | 143 (19.72) | 0.211 |
| ADHD | 35 (3.65) | 6 (2.55) | 29 (4.00) | 0.423 |
| Obesity | 275 (28.65) | 57 (24.25) | 218 (30.07) | 0.170 |
| Hypertension | 214 (22.29) | 52 (22.13) | 162 (22.34) | >0.999 |
| Hyperlipidemia | 206 (21.46) | 53 (22.55) | 153 (21.10) | 0.648 |
| Diabetes Mellitus | 79 (8.23) | 18 (7.66) | 61 (8.41) | 0.768 |
| Fibromyalgia | 13 (1.35) | 2 (0.85) | 11 (1.52) | 0.745 |
| Current/Former Smoker | 297 (30.94) | 76 (32.34) | 221 (30.48) | .0626 |
| *Disease Activity – mean (SD)/median (IQR)* | |  |  |  |
| Tender Joint Count | 2.18 (4.05)/  1 (0-2) | 2.47 (3.82)/  1 (0-3) | 2.08 (4.12)/  0 (0-2) | 0.231/  0.001 |
| Swollen Joint Count | 1.21 (2.84)/  0 (0-1) | 1.61 (3.28)/  0 (0-2) | 1.09 (2.67)/  0 (0-1) | 0.021/  <0.001 |
| % Psoriasis BSA | 1.72 (6.57)/  0.5 (0-1) | 2.12 (6.75)/  1 (0.5-2) | 1.59 (6.52)/  0.5 (0-1) | 0.308/  <0.001 |
| Moderate to Severe PsO^#^--n (%) | 110 (11.46) | 41(17.44) | 69 (9.52) | 0.002 |
| Body Mass Index—mean (SD) | 28.59 (12.55) | 27.55 (5.11) | 28.91 (14.07) | 0.176 |
| Physician Global | 2.36 (1.67)/  2 (1-3) | 2.69 (1.68)/  2.5 (1.5-4) | 2.25 (1.66)/  2 (1-3) | 0.001/  <0.001 |
| RAPID 3—mean (SD) | 9.78 (6.11) | 8.8 (4.99) | 10.18 (6.47) | 0.060 |
| Characteristics | **All**  **(n=960)** | **No b/tsDMARD exposure (n=235)** | **+ b/tsDMARD exposure (n=725)** | **p-value** |
| Erythrocyte Sedimentation Rate | 20.73 (20.25)/ 14 (6-29) | 17.9 (18.20)/  11 (4-26.5) | 21.5 (20.73)/  15 (7-30) | 0.120/  0.060 |
| C-Reactive Protein | 4.63 (7.30)/  14 (6-29) | 5.22 (7.71)/  2.5 (0.8-6) | 4.47 (7.19)/  1.9 (0.7-5.1) | 0.363/  0.430 |
| Current Use of csDMARD^X^ - n(%) | 312 (32.50) | 110 (46.81) | 202 (27.86) | <.001 |

* Patients may identify as more than one race/ethnicity.

^ PsO skin psoriasis, PsA psoriatic arthritis, ADHD attention deficit hyperactivity disorder, BSA body surface area.

^#^ Moderate to severe PsO is BSA ≥3%.

^X^ csDMARD also includes PDE4 inhibitors

**Table S2. Survival estimates by b/tsDMARD exposure**

|  | **First b/tsDMARD**  **(n=529)** | **Second b/tsDMARD**  **(n=192)** | **Third b/tsDMARD**  **(n=98)** | **Fourth + b/tsDMARD**  **(n=103)** |
| --- | --- | --- | --- | --- |
| **Year 1** | 72.4% | 51.0% | 46.9% | 32.0% |
| **Year 2** | 57.5% | 30.7% | 17.3% | 9.7% |
| **Year 3** | 45.2% | 18.7% | 18.7% | 4.9% |
| Month Estimate | 22.9 (21.7-24.0) | 14.9 (13.1-16.6) | 12.6 (10.5-14.8) | 10.1 (8.3-11.8) |

*Month estimates shown as mean estimate with 95% confidence interval.*

*Fourth+ b/tsDMARD includes fourth, fifth, and sixth line medications.*

**Table S3. Hazard ratios for first b/tsDMARD persistence**

|  | **Year 1** | | | **Year 3** | | |
| --- | --- | --- | --- | --- | --- | --- |
|  | **HR** | **95%CI** | **p-value** | **HR** | **95%CI** | **p-value** |
| ***Unadjusted*** | | | | | | |
| Female Sex | **1.74** | **1.25-2.42** | **0.001** | **1.64** | **1.30-2.07** | **<0.001** |
| Age | 1.01 | 0.99-1.01 | 0.728 | 1.00 | 0.99-1.01 | 0.511 |
| Depression | 1.35 | 0.91-1.98 | 0.135 | 1.26 | 0.95-1.68 | 0.105 |
| Obesity | 1.04 | 0.73-1.50 | 0.814 | 1.16 | 0.90-1.50 | 0.265 |
| Smoker^+^ | 1.12 | 0.85-1.69 | 0.315 | 1.21 | 0.95-1.55 | 0.124 |
| Axial Involvement | 1.06 | 0.72-1.55 | 0.782 | 1.10 | 0.83-1.44 | 0.505 |
| Dactylitis | 0.95 | 0.66-1.36 | 0.774 | 1.00 | 0.78-1.29 | 0.975 |
| Peripheral Erosions | 0.96 | 0.66-1.39 | 0.839 | 0.99 | 0.76-1.28 | 0.942 |
| ***Adjusted**** | | | | | | |
| Female Sex | **1.49** | **1.05-2.11** | **0.025** | **1.48** | **1.16-1.89** | **0.002** |
| Age | 1.00 | 0.99-1.01 | 0.808 | 1.00 | 0.99-1.01 | 0.417 |
| Depression | 1.26 | 0.84-1.89 | 0.273 | 1.19 | 0.89-1.60 | 0.245 |
| Obesity | 0.99 | 0.68-1.42 | 0.966 | 1.09 | 0.85-1.41 | 0.502 |
| Smoker^+^ | 1.22 | 0.85-1.75 | 0.285 | 1.25 | 0.77-1.62 | 0.089 |
| Axial Involvement | 1.04 | 0.70-1.55 | 0.840 | 1.04 | 0.78-1.38 | 0.793 |
| Dactylitis | 0.87 | 0.59-1.29 | 0.485 | 0.96 | 0.73-1.26 | 0.775 |
| Peripheral Erosions | 0.93 | 0.63-1.38 | 0.722 | 0.96 | 0.73-1.27 | 0.778 |

^+^ Smoker includes current and former smokers.

* Adjusted for sex, depression, obesity, and smoking status.

**Table S4. Regression models for multi-b/tsDMARD failure vs 1 b/tsDMARD exposure**

| **Factor** | **Odds Ratios** | **95%CI** | **p-value** | **Model** |
| --- | --- | --- | --- | --- |
| *Unadjusted* | | | | |
| Age | 1.03 | 1.01-1.04 | <0.001 | -1.836+0.27(age) |
| Female Sex | 1.93 | 1.30-2.85 | 0.001 | -.253 +.766(sex) |
| Disease Duration | 1.06 | 1.04-1.09 | <0.001 | -1.108+ .60(disease duration) |
| Axial Involvement | 1.89 | 1.16-3.00 | 0.011 | -.311+ .622(axial) |
| Depression | 2.15 | 1.31-3.53 | 0.003 | -.253+ .766(depression) |
| Anxiety | 1.36 | 0.84-2.21 | 0.215 | -.399+ .308(anxiety) |
| Hypertension | 2.12 | 1.35-3.35 | 0.001 | -.295+ .753(hypertension) |
| Hyperlipidemia | 1.35 | 0.84-2.18 | 0.215 | -.405+.301 (hyperlipidemia) |
| Diabetes | 2.92 | 1.18-4.46 | 0.015 | -.157+ .839(diabetes) |
| Smoking* | 1.23 | 0.81-1.87 | 0.326 | -.454+.209(smoker) |
| Obesity | 1.63 | 1.07-2.49 | 0.023 | -3.94+ .488(obesity) |
| Moderate to Severe Skin Severity | 1.74 | 0.91-3.33 | 0.095 | .228+ .553(moderate-severe skin activity) |
| *Adjusted Analysis^+^* | | | | |
| Age | 1.02 | 0.99-1.03 | 0.107 | -1.157+ .060(disease duration)+ .015(age)+ .849(sex)+ .701(depression)+ .040 (smoker)+ .528 (obesity)+ .764 (moderate to severe skin severity) |
| Female Sex | 2.34 | 1.44-3.81 | <0.001 | -1.157+ .060(disease duration)+ .015(age)+ .849(sex)+ .701(depression)+ .040 (smoker)+ .528 (obesity)+ .764 (moderate to severe skin severity) |
| Disease Duration | 1.06 | 1.03-1.09 | <0.001 | -1.157+ .060(disease duration)+ .015(age)+ .849(sex)+ .701(depression)+ .040 (smoker)+ .528 (obesity)+ .764 (moderate to severe skin severity) |
| Axial Involvement | 2.06 | 1.17-3.61 | 0.012 | -1.038+ .059(disease duration)+ .016(age)+ .847(sex)+ .669(depression)+ .047(smoker)+ .567 (obesity)+ .661(moderate to severe skin severity)+ .722(axial) |
| Depression | 2.02 | 1.10-3.71 | 0.024 | -1.157+ .060(disease duration)+ .015(age)+ .849(sex)+. 701(depression)+ .040 (smoker)+ .528 (obesity)+ .764 (moderate to severe skin severity) |
| Anxiety | 0.93 | 0.48-1.80 | 0.83 | -1.150+ .060(disease duration)+ .015(age)+ .853(sex)+.732 (depression)+ .050(smoker)+ .528(obesity)+ .768(moderate to severe skin severity) - .72(anxiety) |
| Hypertension | 1.45 | 0.79-2.66 | 0.237 | -.887+ .059(disease duration)+ 0.11(age)+ .851(sex)+ .661(depression)+ .041(smoker)+ .494(obesity)+ .757(moderate to severe skin severity) + .369(hypertension) |
| Hyperlipidemia | 0.84 | 0.42-1.56 | 0.536 | -1.325+ .060(disease duration)+ .017(age)+ .850(sex)+ .706(depression)+ .052(smoker)+ .518(obesity)+ .755(moderate to severe skin severity) - .206(hyperlipidemia) |
| Diabetes | 1.28 | 0.58-2.92 | 0.565 | -1.023+ .059(disease duration)+ .014(age)+ .839(sex)+ .717(depression)+ .028 (smoker)+ .508(obesity)+ .741 (moderate to severe skin severity) + .244(diabetes) |
| Smoking* | 1.04 | 0.61-1.77 | 0.881 | -1.157+ .060(disease duration)+ .015(age)+ .849(sex)+. 701(depression)+ .040 (smoker)+ .528 (obesity)+ .764 (moderate to severe skin severity) |
| Obesity | 1.7 | 1.02-2.82 | 0.042 | -1.157+ .060(disease duration)+ .015(age)+ .849(sex)+. 701(depression)+ .040 (smoker)+ .528 (obesity)+ .764 (moderate to severe skin severity) |
| Moderate to Severe Skin Severity | 2.15 | 0.96-4.78 | 0.061 | -1.157+ .060(disease duration)+ .015(age)+ .849(sex)+. 701(depression)+ .040 (smoker)+ .528 (obesity)+ .764 (moderate to severe skin severity) |

*Smoking includes current and former smokers.

^+^ Multivariate analysis adjusts for age, sex, disease duration, depression, smoking, obesity, and skin severity.

**Table S5. Baseline characteristics of patients requiring 1 b/tsDMARD compared to multi-b/tsDMARD failure patients (≥ 5 years of disease duration)**

| Characteristics | All  (n=299) | 1 b/tsDMARD^O^  (n=146) | 4+ b/tsDMARD^O^  (n=153) | p-value |
| --- | --- | --- | --- | --- |
| *Demographics* |  |  |  |  |
| Age- median (IQR) | 51 (40-62) | 47.5 (39-59) | 55 (40-63.5) | 0.040 |
| Female- n (%) | 153 (47.22) | 62 (42.47) | 91 (59.48) | 0.004 |
| Race/Ethnicity- n (%)* |  |  |  |  |
| Asian | 24 (7.41) | 15 (10.27) | 9 (5.88) |  |
| Black | 4 (1.23) | 2 (1.37) | 2 (1.31) |  |
| White | 256 (79.01) | 123 (84.25) | 133 (86.93) |  |
| Other | 15 (4.63) | 6 (4.11) | 9 (5.88) |  |
| Hispanic | 19 (5.86) | 7 (4.79) | 12 (7.84) | 0.340 |
| *Psoriatic Disease and Treatment Timeline – median (IQR))* | | | |  |
| Age PsO Onset^ | 23 (15-35) | 25 (18-35) | 22 (14-35) | 0.236 |
| Age PsA Onset | 36 (27-48) | 35 (26-45) | 38 (28-48.5) | 0.163 |
| PsO to PsA Transition (years) | 9 (1-17.75) | 7 (1-14) | 9 (2-21) | 0.070 |
| PsA Diagnosis Delay (years) | 0 (0-2) | 0 (0-2) | 0 (0-2) | 0.845 |
| Disease Duration (years) | 11 (7-17) | 10 (7-16) | 11 (6-19) | 0.994 |
| Time to First Biologic (years) | 1 (0-5) | 2 (0-6) | 0.5 (0-2) | <.001 |
| Number of b/tsDMARDs | 4 (1-5) | 1 (1-1) | 5 (4-6) |  |
| Number of MOAs | 1 (1-3) | 1 (1-1) | 3 (2-3) |  |
| csDMARD^X^ use ever—n(%) | 212 (70.90) | 91 (62.33) | 121 (79.08) | 0.002 |
| *Psoriatic Disease Phenotype- n (%)* | |  |  |  |
| Imaging Erosions | 83 (25.62) | 35 (23.97) | 48 (31.37) | 0.158 |
| Peripheral Deformities | 31 (9.57) | 19 (13.01) | 12 (7.84) | 0.184 |
| Enthesitis | 92 (28.40) | 32 (21.92) | 60 (39.22) | 0.001 |
| Dactylitis | 84 (28.09) | 37 (25.34) | 47 (30.72) | 0.307 |
| Axial Disease | 63 (19.44) | 23 (15.75) | 40 (26.14) | 0.033 |
| Scalp Psoriasis | 176 (54.32) | 88 (60.27) | 88 (57.52) | 0.640 |
| Inverse Psoriasis | 52 (16.05) | 26 (17.81) | 26 (16.99) | 0.880 |
| Nail Involvement | 144 (44.44) | 65 (44.52) | 79 (51.63) | 0.247 |
| *Comorbidities- n(%)* |  |  |  |  |
| Uveitis | 10 (3.09) | 5 (3.42) | 5 (3.27) | >0.999 |
| Inflammatory Bowel Disease | 8 (2.47) | 1 (0.68) | 7 (4.58) | 0.067 |
| Depression | 57 (17.59) | 20 (13.70) | 37 (24.18) | 0.027 |
| Anxiety | 58 (17.90) | 23 (15.75) | 35 (22.88) | 0.144 |
| ADHD | 11 (3.40) | 4 (2.74) | 7 (4.58) | 0.542 |
| Obesity | 80 (26.76) | 24 (16.44) | 56 (36.60) | <0.001 |
| Hypertension | 68 (20.99) | 23 (15.75) | 45 (29.41) | 0.006 |
| Hyperlipidemia | 62 (19.14) | 30 (20.55) | 32 (20.92) | >0.999 |
| Diabetes Mellitus | 28 (8.64) | 8 (5.48) | 20 (13.07) | 0.029 |
| Fibromyalgia | 4 (1.23) | 1 (0.68) | 3 (1.96) | 0.623 |
| Current/Former Smoker | 92 (30.77) | 44 (30.14) | 48 (31.37) | 0.900 |
| *Disease Activity – mean (SD)/ median (IQR)* | |  |  |  |
| Tender Joint Count | 1.91 (3.85)/  0 (0-2) | 1.18 (3.33)/  0 (0-1) | 2.63 (4.18)/  1 (0-3) | 0.001/ <0.001 |
| Swollen Joint Count | 1.04 (2.53)/  0 (0-1) | 0.62 (1.80)/  0 (0-0) | 1.47 (3.04)/  0 (0-1.5) | 0.003/ <0.001 |
| % Psoriasis BSA | 1.13 (3.20)/  0.5 (0-1) | 0.73 (1.53)/  0.5 (0-1) | 1.51 (4.17)/  0.5 (0-1) | 0.021/ 0.158 |
| Moderate to Severe PsO^#^ | 26 (8.70) | 7 (4.79) | 9 (5.88) | 0.024 |
| Body Mass Index—mean (SD) | 28.04 (6.68) | 26.66 (6.11) | 29.3 (6.94) | <0.001 |
| Physician Global | 2.19 (1.64)/  2 (1-3) | 1.56 (1.44)/  1 (0.63-2) | 2.73 (1.62)/  2.5 (1.5-3.5) | <0.001/ <0.001 |
| Characteristics | **All**  **(n=299)** | **1 b/tsDMARD^O^**  **(n=146)** | **4+ b/tsDMARD**  **(n=153)** | **p-value** |
| RAPID 3—mean(SD) | 10.45 (6.26) | 7.1 (4.72) | 12.81 (6.16) | <0.001 |
| Erythrocyte Sedimentation Rate | 22.78 (21.48)/  18 (7.25-33) | 14.1 (13.52)/  8 (5-20.5) | 29.68 (24.06)/  23 (13.75-36.25) | <0.001/ <0.001 |
| C-Reactive Protein | 4.78 (6.99)/  1.8 (0.63-5.08) | 3.16 (4.58)/  1.3 (0.5-3.15) | 6.15 (8.30)/  2.4 (1-6.94) | 0.005/ 0.007 |
| Current Use of csDMARD- n(%) | 87 (26.85) | 27 (18.49) | 60 (39.22) | 0.013 |

^O^ All patients in this table have had psoriatic arthritis for at least 5 years.

* Patients may identify as more than one race/ethnicity.

^ PsO skin psoriasis, PsA psoriatic arthritis, MOA mechanism of action, ADHD attention deficit hyperactivity disorder, BSA body surface area.

^X^ csDMARD also includes PDE4 inhibitors.

^#^ Moderate to severe PsO is BSA ≥3%.

**Table S6. Regression models for multi-b/tsDMARD failure vs. 1 b/tsDMARD exposure (with ≥5 years of disease duration)**

| **Factor** | **Odds Ratios** | **95%CI** | **p-value** | **Model** |
| --- | --- | --- | --- | --- |
| *Unadjusted Analysis* | | | | |
| Age | 1.02 | 1.00-1.03 | 0.048 | -.786+ .016(age) |
| Female Sex | 1.99 | 1.26-3.15 | 0.003 | .040+ .687(sex) |
| Disease Duration | 1.01 | 0.99-1.04 | 0.332 | -.114+ 0.12(disease duration) |
| Axial Involvement | 1.83 | 1.07-3.36 | 0.029 | .234+ .638(axial) |
| Depression | 2.01 | 1.10-3.66 | 0.023 | .266+ .698(depression) |
| Anxiety | 1.59 | 0.89-2.84 | 0.121 | .189+ .461(anxiety) |
| Hypertension | 2.23 | 1.27-3.92 | 0.005 | .271+.801(hypertension) |
| Hyperlipidemia | 1.02 | 0.58-1.79 | 0.938 | .053+ .022(hyperlipidemia) |
| Diabetes | 2.59 | 1.11-6.00 | 0.029 | .440+ .953(diabetes) |
| Smoking* | 1.06 | 0.65- 1.73 | 0.817 | .058+0.58(smoker) |
| Obesity | 2.83 | 1.62-4.92 | <0.001 | .327+ 1.041(obesity) |
| Moderate to Severe Skin Severity | 2.78 | 1.13-6.85 | 0.026 | .487+ 1.023(moderate to severe skin activity) |
| *Adjusted Analysis^+^* | | | | |
| Age | 1.017 | 0.99-1.04 | 0.104 | .069+ .017(age)+ .562(sex)+ .007(disease duration)+ .733 (depression)+ .039(smoker)+ .833(obesity)+ 1.239(skin severity) |
| Female Sex | 1.754 | 1.02-3.01 | 0.041 | .069+ .017(age)+ .562(sex)+ .007(disease duration)+ .733 (depression)+ .039(smoker)+ .833(obesity)+ 1.239(skin severity) |
| Disease Duration | 1.007 | 0.98-1.04 | 0.64 | .069+ .017(age)+ .562(sex)+ .007(disease duration)+ .733 (depression)+ .039(smoker)+ .833(obesity)+ 1.239(skin severity) |
| Axial Involvement | 2.176 | 1.14-4.14 | 0.018 | .192+ .018(age)+ .587(sex)+ .008(disease duration)+ .677(depression)+ .057(smoker)+ .885(obesity)+ 1.142(skin severity)+ .92(axial) |
| Depression | 2.081 | 1.06-4.08 | 0.033 | .069+ .017(age)+ .562(sex)+ .007(disease duration)+ .733 (depression)+ .039(smoker)+ .833(obesity)+ 1.239(skin severity) |
| Anxiety | 1.076 | 0.51-2.26 | 0.846 | .059+ .017(age)+ .558(sex)+ .007(disease duration)+ .704(depression)+ .026(smoker)+ .833(obesity)+ 1.235(obesity)+ 1.235(moderate to severe skin severity)+ .074(anxiety) |
| Hypertension | 1.66 | 0.84-3.27 | 0.017 | -.235 + .021(age)+ .562(sex)+ .008(disease duration)+ .747(depression)+ .061(smoker)+ .825(obesity)+ 1.222(obesity)+ 1.222(moderate to severe skin severity)- .357(hyperlipidemia) |
| Hyperlipidemia | 0.699 | 0.35-1.41 | 0.315 | .059+ .017(age)+ .558(sex)+ .007(disease duration)+ .704(depression)+ .026(smoker)+ .833(obesity)+ 1.235(obesity)+ 1.235(moderate to severe skin severity)+ .074(anxiety) |
| Diabetes | 1.664 | 0.61-4.53 | 0.319 | .386+ .015(age)+ .526(sex)+ .006(disease duration)+ .772(depression)+ .007(smoker)+ .793(obesity)+ .793(obesity)+ 1.249(moderate to severe skin severity)+ .509(diabetes) |
| Smoking* | 1.039 | 0.58-1.85 | 0.894 | .069+ .017(age)+ .562(sex)+ .007(disease duration)+ .733 (depression)+ .039(smoker)+ .833(obesity)+ 1.239(skin severity) |
| Obesity | 2.3 | 1.27-4.18 | 0.006 | .069+ .017(age)+ .562(sex)+ .007(disease duration)+ .733 (depression)+ .039(smoker)+ .833(obesity)+ 1.239(skin severity) |
| Moderate to Severe Skin Severity | 3.453 | 1.27-9.42 | 0.016 | .069+ .017(age)+ .562(sex)+ .007(disease duration)+ .733 (depression)+ .039(smoker)+ .833(obesity)+ 1.239(skin severity) |

*Smoking includes current and former smokers.

^+^ Adjusted for age, sex, disease duration, depression, smoking, obesity, and skin severity.

**Table S7. Baseline characteristics of patients requiring 1 b/tsDMARD compared to those requiring 3+ b/tsDMARDs**

| Characteristics | All  (n=560) | 1 b/tsDMARD  (n=272) | 3+ b/tsDMARD (n=288) | p-value |
| --- | --- | --- | --- | --- |
| *Demographics* |  |  |  |  |
| Age- mean (SD) | 48 (37-60) | 45 (35-59) | 51 (38.25-61.75) | 0.005 |
| Female- n (%) | 272 (48.57) | 118 (43.38) | 154 (53.47) | 0.022 |
| Race/Ethnicity- n (%)* |  |  |  | 0.072 |
| Asian | 46 (8.21) | 30 (11.03) | 16 (5.56) |  |
| Black | 9 (1.61) | 5 (1.84) | 4 (1.39) |  |
| White | 469 (83.75) | 220 (80.88) | 248 (86.11) |  |
| Other | 36 (6.43) | 16 (5.88) | 20 (6.94) |  |
| Hispanic | 42 (7.50) | 19 (6.99) | 23 (7.99) | 0.216 |
| *Psoriatic Disease and Treatment Timeline – median (IQR)* | | | |  |
| Age PsO Onset^ | 24 (16-35) | 25.5 (18-35.75) | 22 (14-35) | 0.031 |
| Age PsA Onset | 36 (27.75-48) | 36 (27-47) | 37 (28-48) | 0.905 |
| PsO to PsA Transition (years) | 8 (2-17) | 8 (2-15) | 9 (2-20.5) | 0.189 |
| PsA Diagnosis Delay (years) | 1 (0-2) | 1 (0-2) | 0 (0-2) | 0.080 |
| Disease Duration (years) | 8 (4-14) | 6 (3-12) | 9 (5-17) | <0.001 |
| Time to First Biologic (years) | 1 (0-3) | 1 (0-3) | 0 (0-2) | 0.155 |
| Year of First Biologic | 2016  (2011-2019) | 2016  (2013.75-2020) | 2014  (2009-2018) | <0.001 |
| Number of b/tsDMARDs | 3 (1-4) | 1.00 (0.00) | 4 (3-5) |  |
| Number of MOAs | 1 (1-2) | 1.00 (0.00) | 2 (2-3) |  |
| csDMARD^X^ use ever—n(%) | 343 (61.25) | 141 (51.84) | 202 (70.14) | <0.001 |
| *Psoriatic Disease Phenotype- n (%)* | |  |  |  |
| Imaging Erosions | 134 (23.93) | 60 (22.06) | 6 (2.08) | 0.427 |
| Peripheral Deformities | 44 (7.86) | 29 (10.66) | 15 (5.21) | 0.018 |
| Enthesitis | 201 (35.89) | 85 (31.25) | 116 (40.28) | 0.048 |
| Dactylitis | 159 (28.39) | 73 (26.84) | 86 (29.86) | 0.454 |
| Axial Disease | 108 (19.29) | 42 (15.44) | 65 (22.57) | 0.041 |
| Scalp Psoriasis | 331 (59.11) | 160 (58.82) | 170 (59.03) | >0.999 |
| Inverse Psoriasis | 87 (15.54) | 42 (15.44) | 45 (15.63) | >0.999 |
| Nail Involvement | 201 (35.89) | 85 (31.25) | 272 (94.44) | 0.048 |
| *Comorbidities- n(%)* |  |  |  |  |
| Uveitis | 19 (3.39) | 7 (2.57) | 12 (4.17) | 0.355 |
| Inflammatory Bowel Disease | 12 (2.14) | 4 (1.47) | 8 (2.78) | 0.385 |
| Depression | 106 (18.93) | 35 (12.87) | 70 (24.31) | <0.001 |
| Anxiety | 112 (20.00) | 46 (16.91) | 66 (22.92) | 0.091 |
| ADHD | 20 (3.57) | 7 (2.57) | 13 (4.51) | 0.259 |
| Obesity | 172 (30.71) | 71 (26.10) | 101 (35.07) | 0.024 |
| Hypertension | 124 (22.14) | 47 (17.28) | 77 (26.74) | 0.008 |
| Hyperlipidemia | 115 (20.54) | 48 (17.65) | 66 (22.92) | 0.142 |
| Diabetes Mellitus | 46 (8.21) | 17 (6.25) | 29 (10.07) | 0.124 |
| Fibromyalgia | 11 (1.96) | 5 (1.84) | 6 (2.08) | >0.999 |
| Current/Former Smoker | 166 (29.64) | 78 (28.68) | 88 (30.56) | 0.644 |
| *Disease Activity – mean (SD)/ median (IQR)* | |  |  |  |
| Tender Joint Count | 2.01 (3.89)/  0 (0-2) | 1.52 (3.44)/  1 (1-1) | 2.49 (4.22)/  1 (0-3) | 0.006/ <0.001 |
| Swollen Joint Count | 1.07 (2.60)/  0 (0-1) | 0.78 (1.95)/  0 (0-0) | 1.35 (3.08)/  0 (0-1) | 0.015/ 0.009 |
| Characteristics | **All**  **(n=560)** | **1 b/tsDMARD**  **(n=272)** | **3+ b/tsDMARD (n=288)** | **p-value** |
| % Psoriasis BSA | 1.39 (4.43)/  0.5 (0-1) | 1.02 (2.02)/  .5 (0-1) | 1.73 (5.80)/  0.5 (0-1) | 0.075/ 0.357 |
| Moderate to Severe PsO^#^ | 55 (8.92) | 20 (7.35) | 35 (12.15) | 0.086 |
| Body Mass Index—mean (SD) | 29.18 (15.55) | 29.16 (21.24) | 29.21 (6.73) | 0.973 |
| Physician Global | 2.25 (1.64)/  2 (1-3) | 1.81 (1.46)/  1.5 (1-2.38) | 2.64 (1.68)/  2.5 (1.5-3.5) | <0.001/ <0.001 |
| RAPID 3—mean (SD) | 10.57 (6.48) | 8.36 (5.74) | 12.35 (6.52) | <0.001 |
| Erythrocyte Sedimentation Rate | 21.64 (20.62)/ 16 (7-31) | 17.46 (17.87)/  10 (5-24) | 25.46 (22.23)/  19 (9-33) | 0.001/ <0.001 |
| C-Reactive Protein | 4.66 (7.80)/  2.1 (0.7-5.1) | 4.05 (8.51)/  1.5 (0.6-4.55) | 5.23 (7.03)/  2.5 (0.85-6.15) | 0.202/ 0.027 |
| Current Use of csDMARD- n(%) | 156 (27.86) | 63 (23.16) | 93 (32.29) | 0.018 |
| Current Use of b/tsDMARD- n(%) | 444 (79.29) | 222 (81.62) | 222 (77.08) | 0.174 |

* Patients may identify as more than one race/ethnicity.

^ PsO skin psoriasis, PsA psoriatic arthritis, ADHD attention deficit hyperactivity disorder, BSA body surface area, MOA mechanism of action.

^X^ csDMARD also includes PDE4 inhibitors.

^#^ Moderate to severe PsO is BSA ≥3%.

**Table S8. Regression models for of patients requiring 3+ b/tsDMARDs compared to those requiring 1 b/tsDMARDs**

| **Factor** | **Odds Ratios** | **95%CI** | **p-value** | **Model** |
| --- | --- | --- | --- | --- |
| *Unadjusted Analysis* | | | | |
| Age | 1.01 | 1.01-1.03 | 0.006 | -.736 + .016(age) |
| Female Sex | 1.5 | 1.08-2.10 | 0.017 | .064+ .405(sex) |
| Disease Duration | 1.05 | 1.05-1.07 | <0.001 | -.391+ .048(disease duration) |
| Axial Involvement | 1.55 | 1.01-3.38 | 0.044 | .193+ .440(axial) |
| Depression | 2.11 | 1.35-3.28 | <0.001 | .293+ .744(depression) |
| Anxiety | 1.46 | 0.96-2.22 | 0.077 | .172+ .379(anxiety) |
| Hypertension | 1.75 | 1.61-2.63 | 0.007 | .215+ .558(hypertension) |
| Hyperlipidemia | 1.35 | 0.90-2.05 | 0.152 | .147+ .302(hyperlipidemia) |
| Diabetes | 1.68 | 0.90-3.13 | 0.103 | .275+ .519(diabetes) |
| Smoking* | 1.09 | 0.76-1.57 | 0.627 | .076+. 090(smoker) |
| Obesity | 1.55 | 1.07-2.25 | 0.020 | .132+ .441(obesity) |
| Moderate to Severe Skin Severity | 1.69 | 0.95-3.02 | 0.077 | .298+ .532(skin severity) |
| *Adjusted Analysis^+^* | | | | |
| Age | 1.009 | 0.99-1.02 | 0.256 | -.135+ .009(age)+ .468(sex) +.047(disease duration)+ .758(depression) +.000(smoker)+ .515(obesity)+ .713(skin severity) |
| Female Sex | 1.598 | 1.07-2.39 | 0.022 | -.135+ .009(age)+ .468(sex) +.047(disease duration)+ .758(depression) +.000(smoker)+ .515(obesity)+ .713(skin severity) |
| Disease Duration | 1.048 | 1.02-1.07 | <0.001 | -.135+ .009(age)+ .468(sex) +.047(disease duration)+ .758(depression) +.000(smoker)+ .515(obesity)+ .713(skin severity) |
| Axial Involvement | 1.56 | 0.95-2.55 | 0.078 | -.008+ .008(age)+ .459(sex) +.046(disease duration)+ .752(depression) - .006(smoker)+ .516(obesity)+ .674(skin severity)+ .443 (axial) |
| Depression | 2.134 | 1.27-3.60 | 0.004 | -.135+ .009(age)+ .468(sex) +.047(disease duration)+ .758(depression) +.000(smoker)+ .515(obesity)+ .713(skin severity) |
| Anxiety | 1.08 | 0.62-1.86 | 0.793 | -.143+ .009(age)+ .464(sex) +.047(disease duration)+ .725(depression) - .008(smoker)+ .514(obesity)+ .708(skin severity)+ .073 (anxiety) |
| Hypertension | 1.29 | 0.75-2.23 | 0.361 | .056+ .006(age)+ .475(sex) +.047(disease duration)+ .738(depression) + .002(smoker)+ .691(obesity)+ .691(skin severity)+ .255 (hypertension) |
| Hyperlipidemia | 0.936 | 0.54-1.62 | 0.814 | -.189+ .009(age)+ .469(sex) +.047(disease duration)+ .759(depression) + .002(smoker)+ .511(obesity)+ .713(skin severity)- .066 (hyperlipidemia) |
| Diabetes | 0.95 | 0.44-2.12 | 0.885 | -.164+ .009(age)+ .469(sex) +.047(disease duration)+ .759(depression) + .002(smoker)+ .511(obesity)+ .713(skin severity)- .066 (hyperlipidemia) |
| Smoking* | 1 | 0.64-1.57 | 0.999 | -.135+ .009(age)+ .471(sex) +.047(disease duration)+ .755(depression) +.002(smoker)+ .520(obesity)+ .719(skin severity)- .056(diabetes) |
| Obesity | 1.674 | 1.09-2.57 | 0.018 | -.135+ .009(age)+ .468(sex) +.047(disease duration)+ .758(depression) +.000(smoker)+ .515(obesity)+ .713(skin severity) |
| Moderate to Severe Skin Severity | 2.04 | 1.03-4.04 | 0.041 | -.135+ .009(age)+ .468(sex) +.047(disease duration)+ .758(depression) +.000(smoker)+ .515(obesity)+ .713(skin severity) |

*Smoking includes current and former smokers.

^+^ Adjusted for age, sex, disease duration, depression, smoking, obesity, and skin severity.

**Table S9. Baseline characteristics of patients by mechanism of action**

| Characteristics | All  (n=517) | 1 MOA  (n=382) | 3+ MOAs  (n=145) | p-value |
| --- | --- | --- | --- | --- |
| *Demographics* |  |  |  |  |
| Age- median (IQR) | 48 (37-60) | 46 (36-60) | 53 (39-62) | 0.047 |
| Female- n (%) | 257 (49.71) | 180 (47.12) | 58 (42.96) | 0.057 |
| Race/Ethnicity- n (%)* | (0.00) |  |  | 0.468 |
| Asian | 47 (9.09) | 38 (9.95) | 9 (6.67) |  |
| Black | 9 (1.74) | 7 (1.83) | 2 (1.48) |  |
| White | 428 (82.79) | 314 (82.20) | 114 (84.44) |  |
| Other | 33 (6.38) | 23 (6.02) | 10 (7.41) |  |
| Hispanic | 363 (70.21) | 351 (91.88) | 12 (8.89) | 0.231 |
| *Psoriatic Disease and Treatment Timeline—median (IQR)* | | | |  |
| Age PsO Onset^ | 25 (17-35) | 25 (18-35) | 22 (14-36.5) | 0.136 |
| Age PsA Onset | 36 (28-48) | 36 (27.75-47) | 38 (28-50) | 0.622 |
| PsO to PsA Transition (years) | 8 (2-15.75) | 7 (2-15) | 10 (1-20.75) | 0.157 |
| PsA Diagnosis Delay (years) | 1 (0-2) | 1 (0-2.75) | 0 (0-2) | 0.032 |
| Disease Duration (years) | 7 (4-14) | 7 (3-13) | 9 (4-17.75) | 0.006 |
| Time to First Biologic (years) | 1 (0-3) | 1 (0-4) | 0 (0-2) | 0.247 |
| Year of First Biologic | 2016  (2011-2019) | 2016  (2011-2019) | 2015  (2010-2019) | 0.270 |
| Number of b/tsDMARDs | 1 (1-3) | 1 (1-2) | 5 (4-6) |  |
| Number of MOAs | 1 (1-3) | 1 (1-1) | 3 (3-4) |  |
| csDMARD^X^ use ever—n(%) | 326 (63.05) | 228 (59.69) | 98 (67.59) | 0.009 |
| *Psoriatic Disease Phenotype- n (%)* | |  |  |  |
| Imaging Erosions | 124 (23.98) | 87 (22.77) | 37 (27.41) | 0.292 |
| Peripheral Deformities | 52 (10.06) | 41 (10.73) | 11 (8.15) | 0.505 |
| Enthesitis | 171 (33.08) | 118 (30.89) | 53 (39.26) | 0.082 |
| Dactylitis | 138 (26.69) | 99 (25.92) | 39 (26.90) | 0.499 |
| Axial Disease | 89 (17.21) | 59 (15.45) | 30 (22.22) | 0.085 |
| Scalp Psoriasis | 307 (59.38) | 229 (59.95) | 78 (57.78) | 0.684 |
| Inverse Psoriasis | 79 (15.28) | 58 (15.18) | 21 (15.56) | 0.890 |
| Nail Involvement | 244 (47.20) | 183 (47.91) | 61 (45.19) | 0.617 |
| *Comorbidities- n(%)* |  |  |  |  |
| Uveitis | 22 (4.26) | 15 (3.93) | 7 (5.19) | 0.620 |
| Inflammatory Bowel Disease | 14 (2.71) | 10 (2.62) | 4 (2.96) | 0.765 |
| Depression | 89 (17.21) | 61 (15.97) | 28 (20.74) | 0.233 |
| Anxiety | 105 (20.31) | 73 (19.11) | 32 (23.70) | 0.264 |
| ADHD | 21 (4.06) | 13 (3.40) | 8 (5.93) | 0.210 |
| Obesity | 153 (29.59) | 107 (28.01) | 46 (31.72) | 0.180 |
| Hypertension | 110 (21.28) | 78 (20.42) | 32 (23.70) | 0.463 |
| Hyperlipidemia | 102 (19.73) | 78 (20.42) | 24 (17.78) | 0.532 |
| Diabetes Mellitus | 41 (7.93) | 26 (6.81) | 15 (11.11) | 0.137 |
| Fibromyalgia | 9 (1.74) | 6 (1.57) | 3 (2.22) | 0.703 |
| Current/Former Smoker | 154 (29.79) | 113 (29.58) | 41 (28.27) | 0.913 |
| *Disease Activity – mean (SD)/ median (IQR)* | |  |  |  |
| Tender Joint Count | 2.08 (4.21)/  0 (0-2) | 1.82 (4.38)/  0 (0-2) | 2.77 (3.95)/  1 (0-4) | 0.034/ <0.001 |
| Swollen Joint Count | 1.11 (2.65)/  0 (0-1) | 0.99 (2.59)/  0 (0-1) | 1.44 (2.80)/  0 (0-2) | 0.115/ 0.030 |
| % Psoriasis BSA | 1.9 (7.66)/  0.5 (0-1) | 1.68 (7.47)/  0.5 (0-1) | 2.53 (8.16)/  0.5 (0-1.5) | 0.296/ 0.347 |
| Moderate to Severe PsO^#^ --n(%) | 57 (11.03) | 35 (9.16) | 22 (15.17) | 0.036 |
| Body Mass Index—mean (SD) | 29.11 (16.21) | 29.05 (18.40) | 29.28 (6.95) | 0.894 |
|  |  |  |  |  |
| Characteristics | **All**  **(n=517)** | **1 MOA**  **(n=382)** | **3+ MOAs**  **(n=145)** | **p-value** |
| Physician Global | 2.23 (1.70)/  2 (1-3) | 2.03 (1.66)/  1.5 (1-3) | 2.74 (1.69)/  2.5 (1.5-4) | <0.001/ <0.001 |
| RAPID 3—mean (SD) | 10.38 (6.67) | 9.32 (6.62) | 13.43 (5.91) | <0.001 |
| Erythrocyte Sedimentation Rate | 21.67 (21.26)/  15 (6-31.5) | 19.62 (19.60)/  13 (5-28) | 26.64 (24.26)/  20 (9-33.75) | 0.017/  0.013 |
| C-Reactive Protein | 4.68 (7.97)/  1.9 (0.7-5.18) | 4.27 (8.00)/  1.75 (0.6-5) | 5.71 (7.83)/  2.35 (1-6.275) | 0.193/ 0.066 |
| Current Use of csDMARD- n(%) | 147 (28.43) | 103 (26.96) | 44 (32.59) | 0.223 |
| Current Use of b/tsDMARD- n(%) | 403 (77.95) | 305 (79.84) | 98 (72.59) | 0.091 |

* Patients may identify as more than one race/ethnicity.

^ PsO skin psoriasis, PsA psoriatic arthritis, ADHD attention deficit hyperactivity disorder, BSA body surface area, MOA mechanism of action.

^X^ csDMARD also includes PDE4 inhibitors.

^#^ Moderate to severe PsO is BSA ≥3%.

**Table S10.** **Disease activity of patients requiring 1 b/tsDMARD compared to muti-b/tsDMARD failure patients (in those currently on b/tsDMARD at time of visit)**

| Disease Activity Measure | All  (n=350) | | 1 b/tsDMARD  (n=222) | | Multi-Failure PsA  (n=128) | | p-value |
| --- | --- | --- | --- | --- | --- | --- | --- |
| Tender Joint Count | 1.62 | (3.58) | 1.28 | (3.36) | 2.23 | (3.88) | 0.026 |
| Swollen Joint Count | 0.84 | (2.21) | 0.58 | (1.64) | 1.33 | (2.93) | 0.005 |
| % Psoriasis BSA | 1.32 | (4.94) | 0.94 | (1.92) | 1.97 | (7.67) | 0.075 |
| Body Mass Index | 29.62 | (18.98) | 29.47 | (23.30) | 29.87 | (7.30) | 0.852 |
| Physician Global | 2.05 | (1.56) | 1.67 | (1.32) | 2.68 | (1.73) | <0.001 |
| RAPID 3 | 10.07 | (6.21) | 8.21 | (5.51) | 12.84 | (6.22) | <0.001 |
| Erythrocyte Sedimentation Rate | 20.85 | (20.01) | 17.24 | (17.48) | 26.93 | (22.53) | <0.001 |
| C-Reactive Protein | 4.14 | (6.18) | 3.47 | (4.92) | 5.33 | (7.84) | 0.024 |
| Current Use of cDMARD- n(%) | 98 | (28.00) | 45 | (20.27) | 53 | (41.41) | <0.001 |
|  |  |  |  |  |  |  |  |

**Figure S1. Reason for discontinuing b/tsDMARD by exposure (including type of primary failure).** (A) First exposure (n= 499), (B) Second exposure (n=325), (C) Third exposure (n=171), (D) Fourth exposure (n=111), and (E) Fifth exposure (n=55).


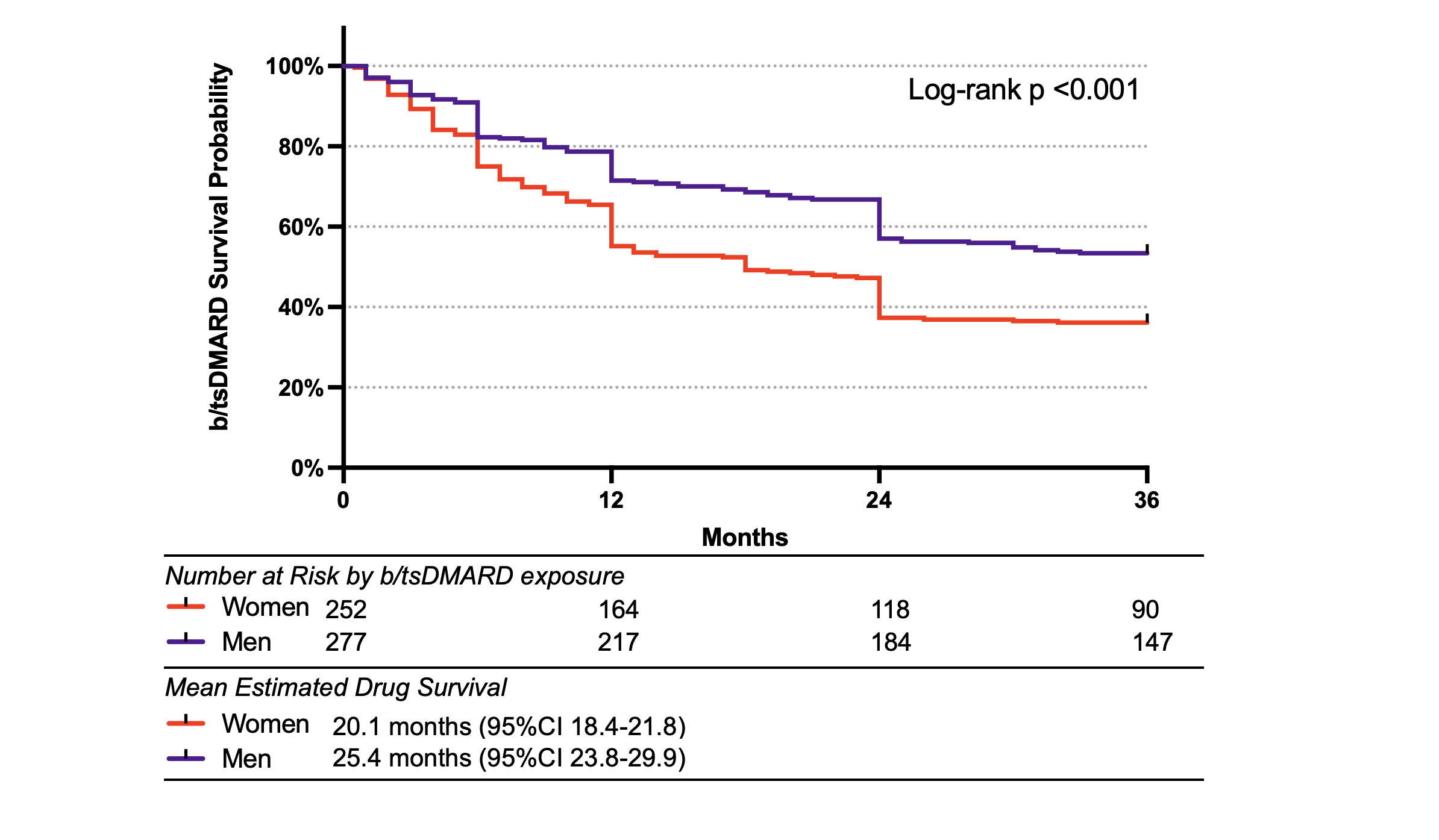


**Figure S2. Kaplan-Meier estimates of first b/tsDMARD persistence by sex.** Persistence was defined as the time from therapy initiation to discontinuation.


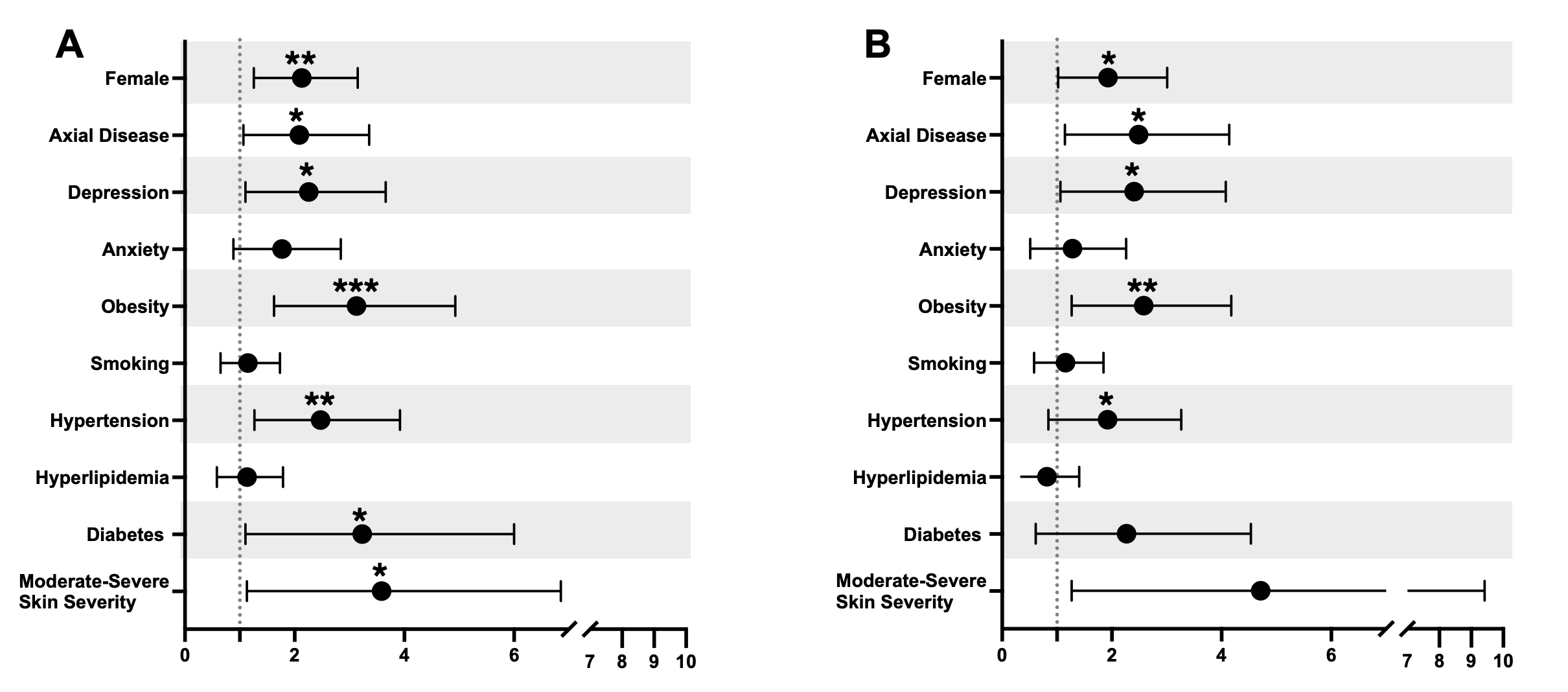


**Figure S3. Risk estimates of multi-b/tsDMARD failure psoriatic arthritis compared to those with 1 b/tsDMARD exposure and at least 5 years of disease**. Odds ratios are unadjusted (A) and adjusted (B) or disease duration, age, sex, depression, smoking status, obesity, and skin severity.


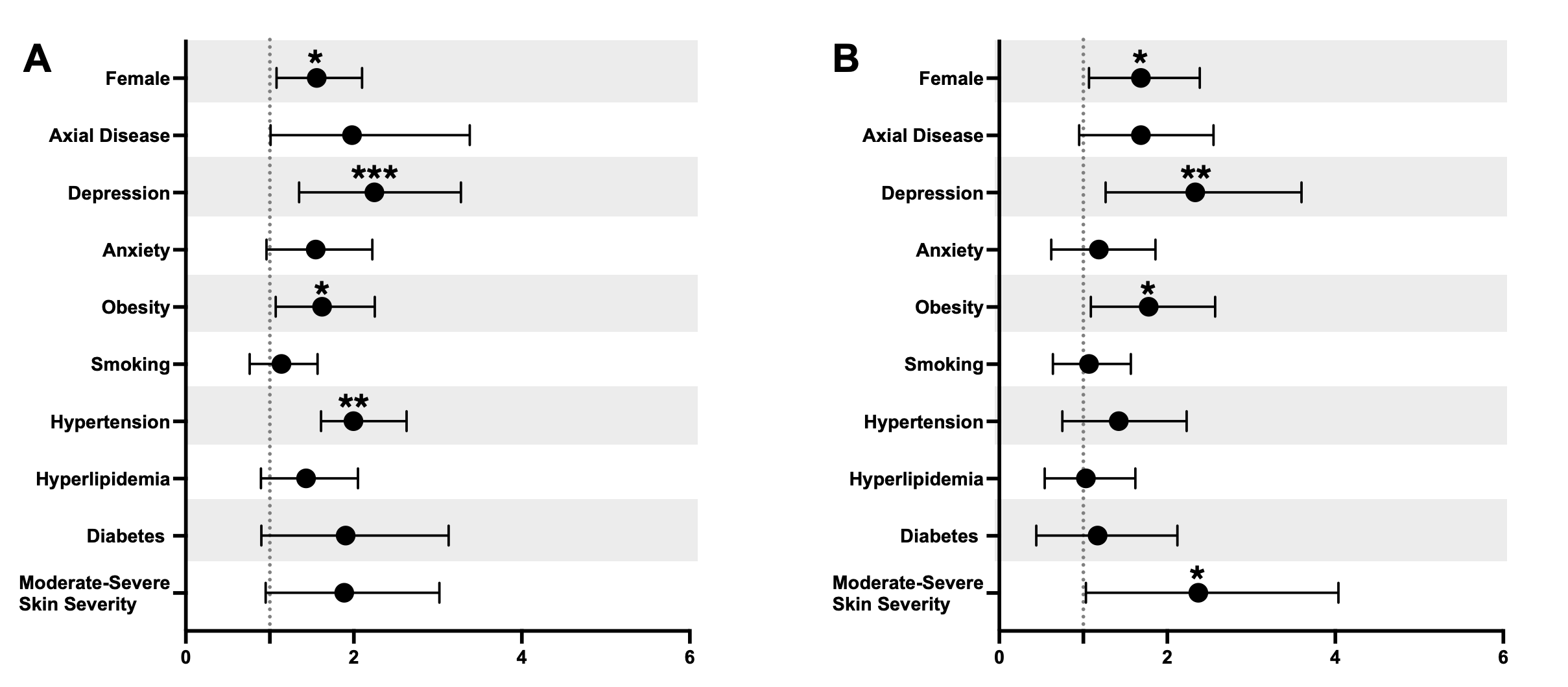


**Figure S4. Risk estimates of requiring 3+ b/tsDMARDs compared to 1 b/tsDMARD**. Odds ratios are unadjusted (A) and adjusted (B) for disease duration, age, sex, depression, smoking status, obesity, and skin severity.
